# FKBP4 is a malignant indicator in luminal A subtype of breast cancer

**DOI:** 10.7150/jca.40982

**Published:** 2020-01-16

**Authors:** Hanchu Xiong, Zihan Chen, Wenwen Zheng, Jing Sun, Qingshuang Fu, Rongyue Teng, Jida Chen, Shuduo Xie, Linbo Wang, Xiao-Fang Yu, Jichun Zhou

**Affiliations:** 1 Department of Surgical Oncology, Sir Run Run Shaw Hospital, Zhejiang University, Hangzhou, 310016, China.; 2 Cancer Institute, Second Affiliated Hospital, School of Medicine, Zhejiang University, Hangzhou, 310016, China.; 3 Surgical Intensive Care Unit, First Affiliated Hospital, Zhejiang University, Hangzhou, Zhejiang, 310016, China.; 4 Rui An Hospital of Traditional Chinese Medicine, Wenzhou, 325200, China.

**Keywords:** FKBP4, luminal A subtype breast cancer, co-expressed genes, bioinformatics analysis

## Abstract

**Purpose**: FKBP4 is a member of the immunophilin protein family, which plays a role in immunoregulation and basic cellular processes involving protein folding and trafficking associated with HSP90. However, the relationship between abnormal expression of FKBP4 and clinical outcome in luminal A subtype breast cancer (LABC) patients remains to be elucidated.

**Methods**: Oncomine, bc-GenExMiner and HPA database were used for data mining and analyzing FKBP4 and its co-expressed genes. GEPIA database was used for screening co-expressed genes of FKBP4.

**Results**: For the first time, we found that higher FKBP4 expression correlated with LABC patients and worse survival. Moreover, the upregulated co-expressed genes of FKBP4 were assessed to be significantly correlated with worse survival in LABC, and might be involved in the biological role of FKBP4.

**Conclusion**: The expression status of FKBP4 is a significant prognostic indicator and a potential drug target for LABC.

## Introduction

Breast cancer (BC) is the most common noncutaneous cancer and the most frequent cause of death in worldwide women 1. Widespread adoption of screening increases breast cancer incidence in given population and current prognostic and predictive biomarkers have markedly improved treatment options for patients. However, BC is a heterogeneous disease of multiple distinct subtypes that differ genetically, pathologically, and clinically 2, it's still necessary to find more reliable markers to further improve therapeutic strategy for individual patients.

The FK506-binding protein 4 (FKBP4, also known as FKBP52) has been reported to possess multiple functions in various kinds of cancers based on its interaction with different cellular targets^3^-^6^. For example, in prostate cancer FKBP4 is found to enhance the transcriptional activity of androgen receptor signaling 3. However, the relationship between abnormal expression of FKBP4 and clinical outcome in luminal A subtype breast cancer (LABC) patients remains unknown. For the first time, we investigated FKBP4 expression in LABC and its interaction with clinicopathological features including molecular subtypes and clinical outcomes by bioinformatics analysis.

In the present study, we used Oncomine, the Human Protein Atlas (HPA) database and breast cancer gene-expression miner (bc-GenExMiner) database to identify the potential difference of FKBP4 expression between BC cancer tissues and adjacent normal samples, as well as the association between FKBP4 and clinical parameters. We further probed into genetic alterations and clinical outcomes of high and low level of FKBP4 expression in breast cancer patients. Lastly, preliminary explorations of the mechanisms of FKBP4 involving BC were carried out by identifying co-expressed genes with a series of online databases.

## Methods

### Data mining and analyzing

The online cancer microarray database, Oncomine (www.oncomine.org) 7 was used to assess the transcription levels of FKBP4 in breast cancer specimens compared with that in normal controls by Students't-test. The immunohistochemistry results of FKBP4 and six co-expressed genes in breast cancer were retrieved from the Human Protein Atlas database (www.proteinatlas.org) 8. The expression and prognostic module of bc-GenExMiner v4.2 (bcgenex.centregauducheau.fr) 9 were used to evaluate the clinicopathological characteristics and prognostic merit of FKBP4 and six co-expressed genes in human breast cancer.

### COSMIC and cBioPortal analysis for mutations

COSMIC database (www.sanger.ac.uk/cosmic/) 10 and cBioPortal database (www.cbioportal.org) 11 were utilized for assessment of FKBP4 mutations.

### Screening co-expressed genes of FKBP4

Co-expressed genes of FKBP4 in breast cancer were collected from GEPIA (gepia.cancerpku.cn) for further evaluation 12.

### Enrichment analysis and pathway annotation

Gene Oncology (GO) and Kyoto Encyclopedia of Genes and Genomes (KEGG) analyses of FKBP4 co-expressed genes were analyzed using The Database for Annotation, Visualization and Integrated Discovery v6.8 (david.ncifcrf.gov)^13^. The String database (www.string-db.org) was applied to construct the protein-protein interaction network for the co-expressed genes identification 14.

## Results

### Upregulated expression of FKBP4 in breast cancer patients

**Figure 1:**
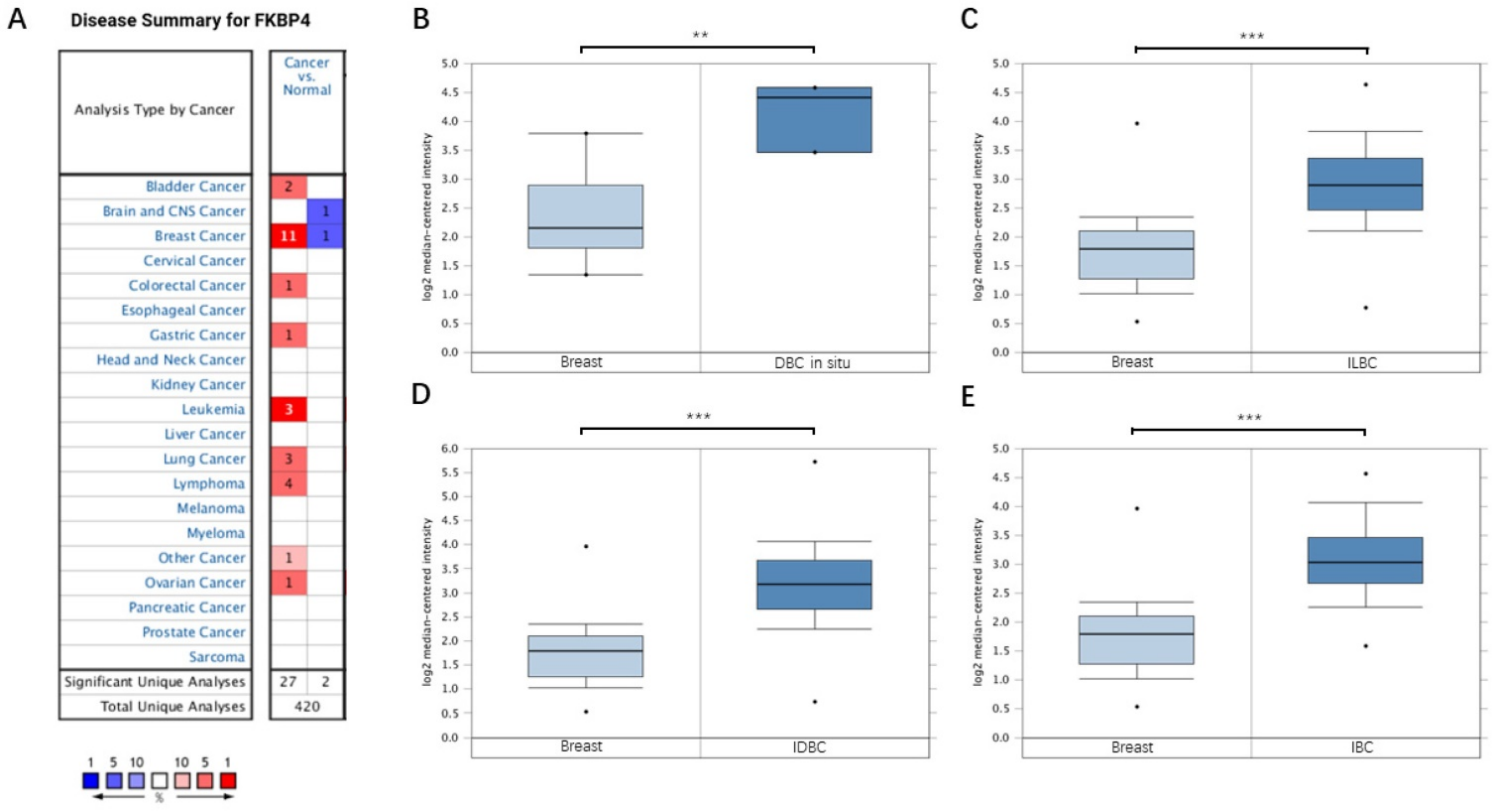
FKBP4 mRNA expression in malignant tumors (Oncomine database). (**A**) The graph is a representation of the datasets with statistically significant mRNA overexpression (red) or reduced expression (blue) of FKBP4 gene (cancer vs normal). Cell color was determined by the best gene rank percentile for the analyses within the cell, and the gene rank was analyzed by percentile of target gene in the top of all genes measured in each research. (**B**) Comparison of FKBP4 mRNA expression between normal breast tissue and DBC. (**C**) Comparison between normal breast tissue and ILBC. (**D**) Comparison between normal breast tissue and IDBC. (**E**) Comparison between normal breast tissue and IBC. *P<0.05, **P<0.01, ***P<0.001.

**Figure 2:**
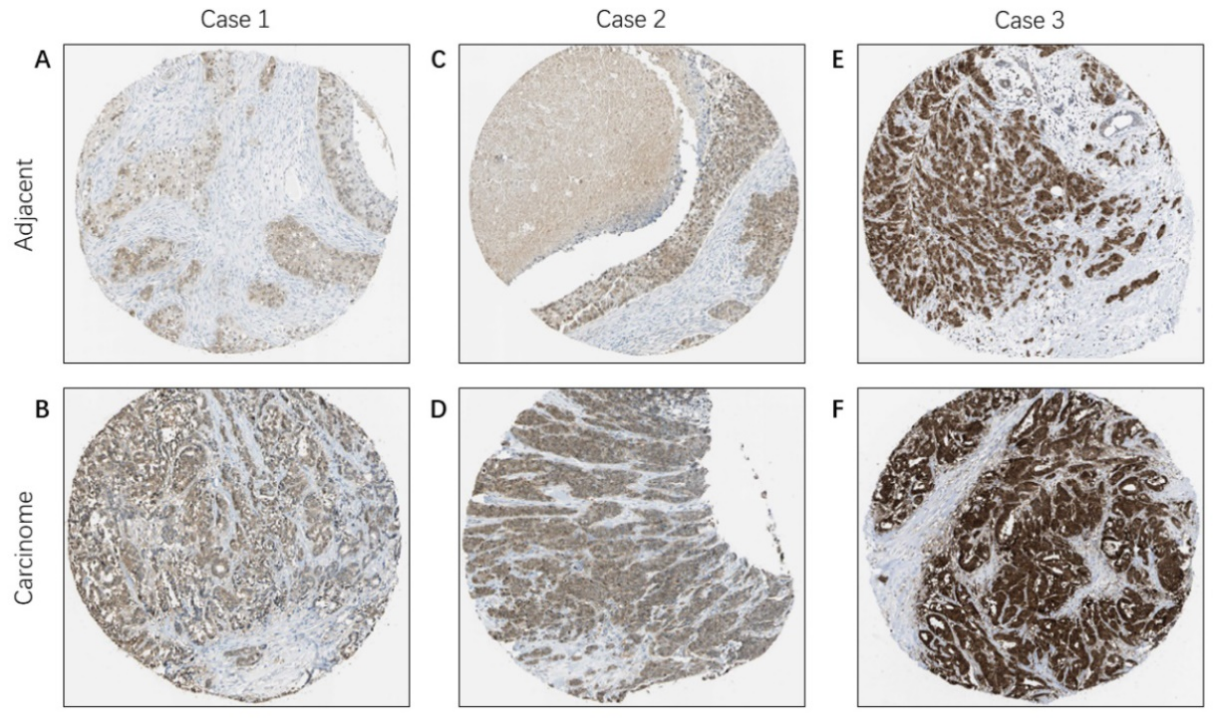
Immunohistochemical staining of FKBP4 protein in BC (HPA database). Representative images of immunohistochemical staining of FKBP4 expression in BC samples and matched adjacent normal tissues.

Based on the Oncomine database, we discovered that FKBP4 mRNA expression was significantly upregulated in cancerous samples compared with normal samples in more than nine types of cancer, including breast cancer, bladder cancer, colorectal cancer, gastric cancer, leukemia and so forth (Figure 1A). Meanwhile, the transcription level of FKBP4 in different types of BC were higher than normal tissues, including ductal breast carcinoma *in situ* (DBC *in situ*) with fold change=3.650, invasive lobular breast carcinoma (ILBC) with fold change=2.245, and invasive ductal breast carcinoma (IDBC) with fold change=2.657, invasive ductal and invasive lobular breast carcinoma (IBC) with fold change=2.480 (Figure 1B-1E). To investigate the protein expression level of FKBP4 in BC, we assessed BC tissue samples and matched adjacent normal tissues from the HPA database. The HPA database indicated that FKBP4 expression was significantly elevated in cancerous tissues compared with corresponding normal tissues when using either antibody HPA006148 (Figure 2A-2D) or antibody CAB017441 (Figure 2E-2F).

### Relationship of FKBP4 with the clinicopathological characteristics and the prognostic merit

**Figure 3:**
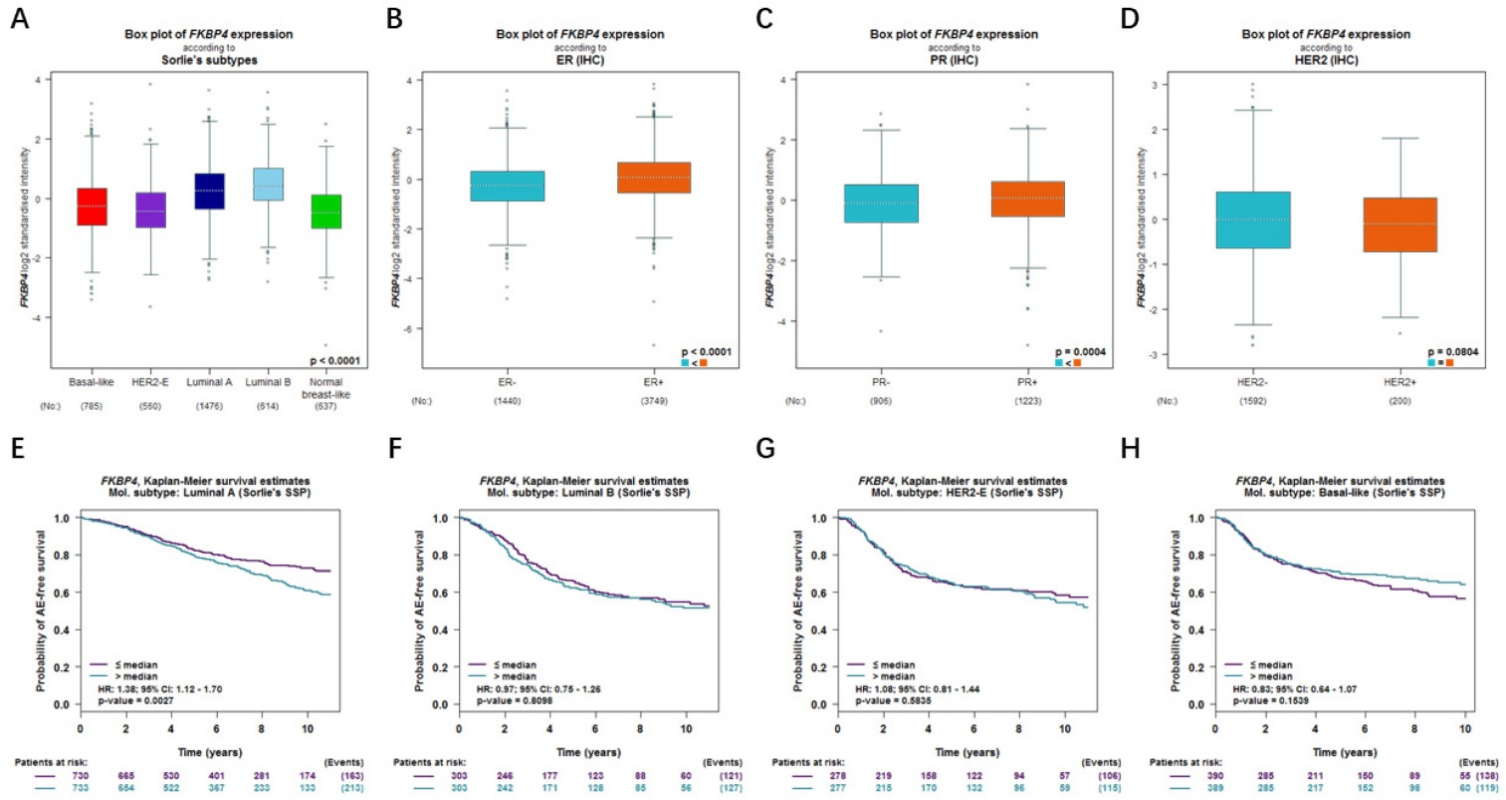
Relationship of FKBP4 with the clinicopathological characteristics and the prognostic merit (bc-GenExMiner v4.2 database). The relationship between mRNA expression of FKBP4 and (**A**) different molecular subtypes, (**B**) ER, (**C**) PR, (**D**) HER2. Survival curves are plotted for patients of (**E**) luminal A, (**F**) luminal B, (**G**) HER2-positive, (**H**) basal-like.

In bc-GenExMiner database, for the molecular subtype, upregulated FKBP4 was significantly related to luminal A, luminal B and basal-like subtype patients than the normal group rather than HER2 positive subtype (Figure 3A). ER and PR status were both positively correlated with FKBP4 expression (Figure 3B-3C). In BC patients with HER2 overexpression, FKBP4 expression has no significant change compared with HER2 negative groups (Figure 3D). To further probe into the correlation of FKBP4 expression and survival, BC patients with diverse molecular subtypes were also investigated. Upregulated FKBP4 was only significantly related to worse survival in luminal A subtype patients (HR=1.38; 95%CI:1.12-1.70, p=0.0027), but not correlated to those in luminal B, HER2 positive and basal-like subtypes of breast cancer patients (HR=0.97; 95%CI:0.75-1.26, p=0.8098; HR=1.08; 95% CI:0.81-1.44, p=0.5835; HR=0.83; 95% CI:0.64-1.07, p=0.1539) (Figure 3E-3H). Taken together, we found that upregulated FKBP4 expression was correlated with poor survival in LABC patients.

### The impact of alterations in FKBP4 gene on the clinical survival

**Figure 4:**
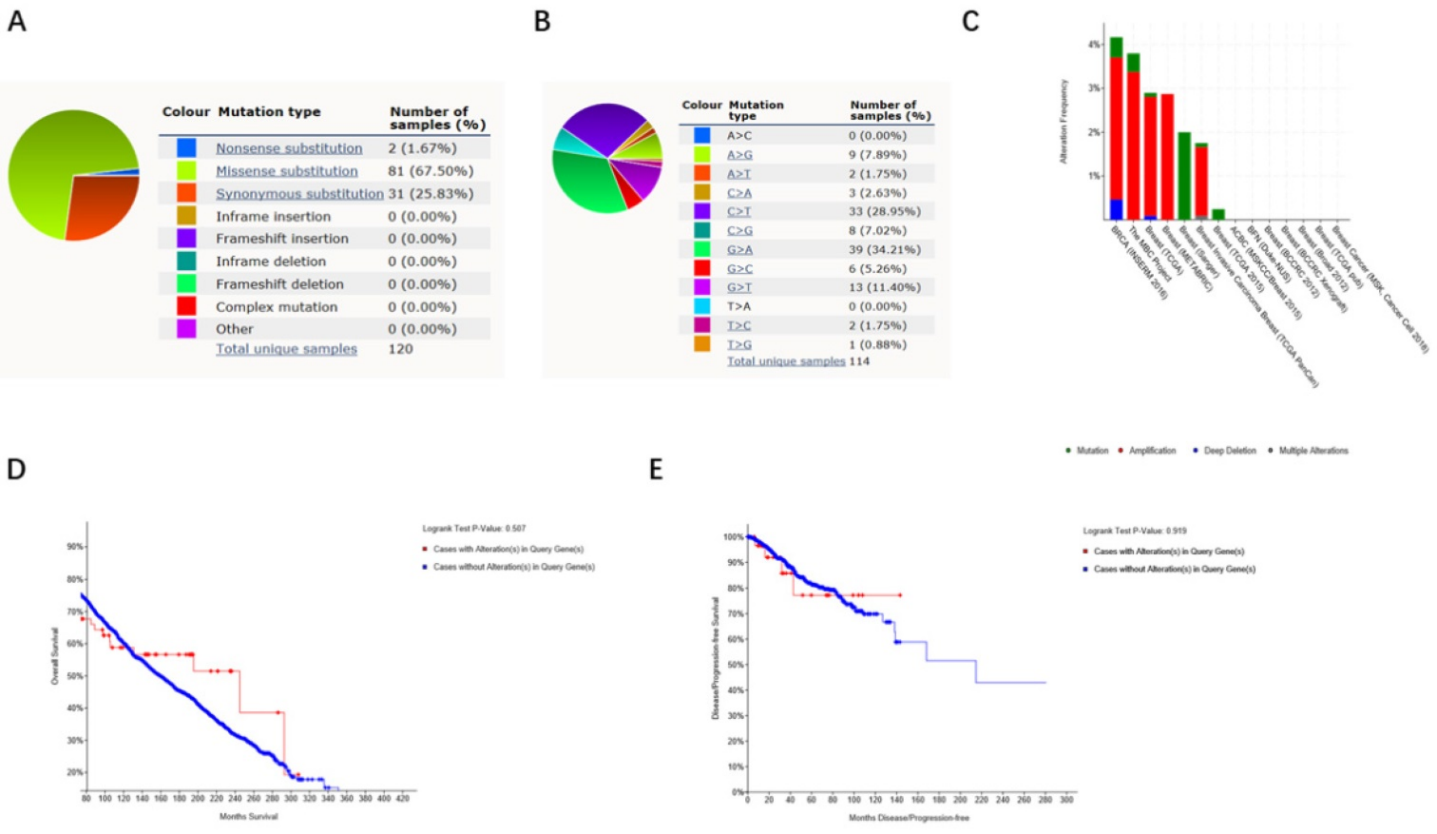
FKBP4 genes expression and mutation analysis in BC (COSMIC and cBioPortal). (**A**,** B**) Pie-chart showed the percentage of the mutation type of FKBP4 in BC according to COSMIC database. (**C**) Oncoprint in cBioPortal represented the proportion and distribution of samples with alterations in FKBP4 gene. (**D**) Kaplan-Meier plots comparing OS in cases with/without FKBP4 gene alterations. (**E**) Kaplan-Meier plots comparing disease free survival (DFS) in cases with/without FKBP4 gene alterations.

By using Catalogue of Somatic Mutations in Cancer (COSMIC), the pie chart described the mutations information including missense substitution, synonymous substitution and frameshift insertion. Missense substitution rate was 67.50%, synonymous substitution rate was 25.83% and nonsense substitution rate was 1.67% of mutant samples of BC. BC mainly had 34.21% G>A, 28.95% C>T and 11.40% G>T mutation in FKBP4 coding strand (Figure 4A-4B). Alteration frequency of FKBP4 mutation in BC was analyzed by using cBioPortal. From 0.25% to 4.25% mutation in the patients with BC was observed (Figure 4C). After analyzed by Kaplan-Meier plot and log-rank test, the alterations in FKBP4 were found no correlations with overall survival (OS) (p=0.507) or disease-free survival (DFS) (p=0.919) in BC patients with/without FKBP4 alterations (Figure 4D-4E).

### Bioinformatics analysis of FKBP4 co-expressed genes

**Figure 5:**
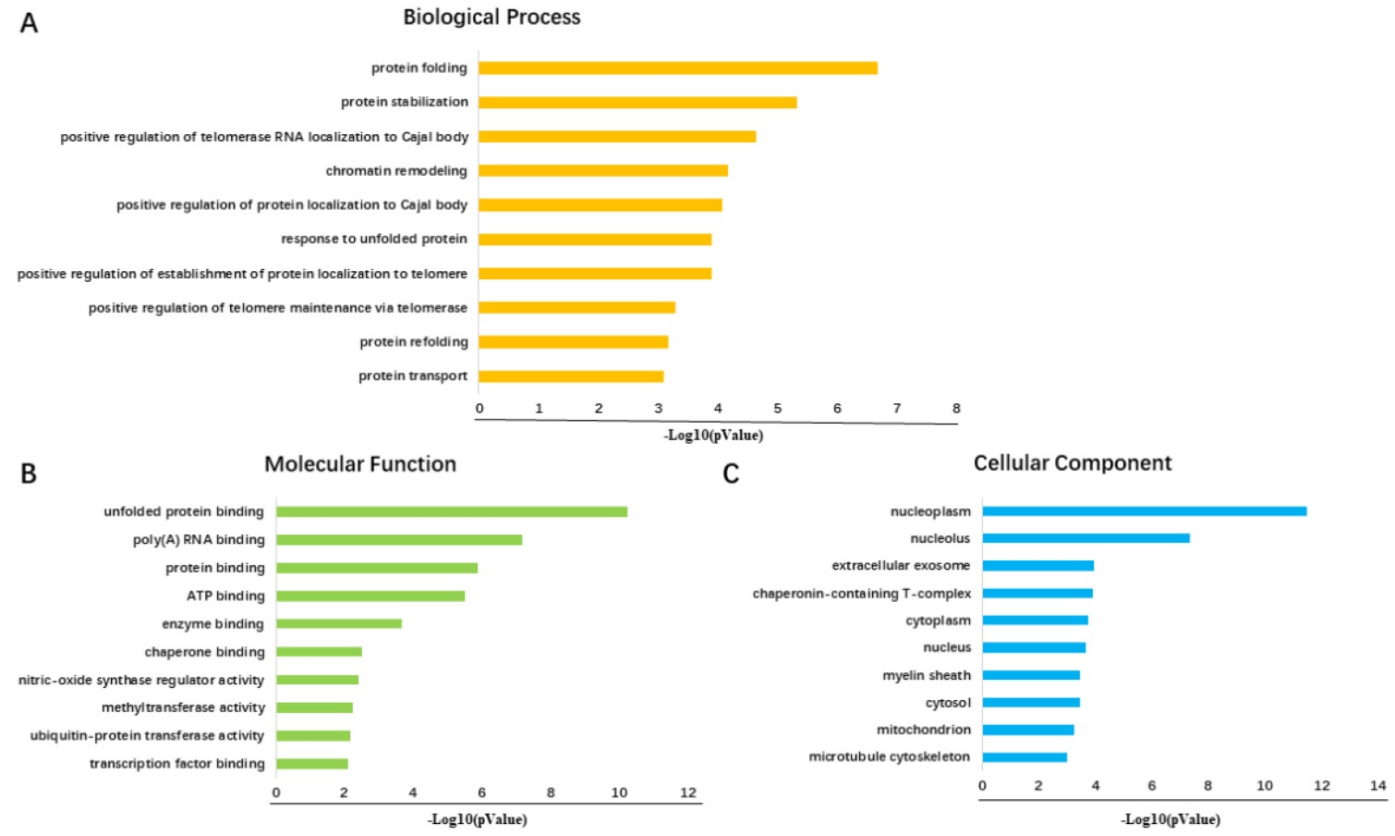
Diagrams of top 10 significant pathways of GO enrichment analysis. (**A**) Graph of the 10 most significant pathways of BP category. (**B**) Top 10 significant terms in the MF category. (**C**) Ten most valuable annotations of the CC category.

**Figure 6:**
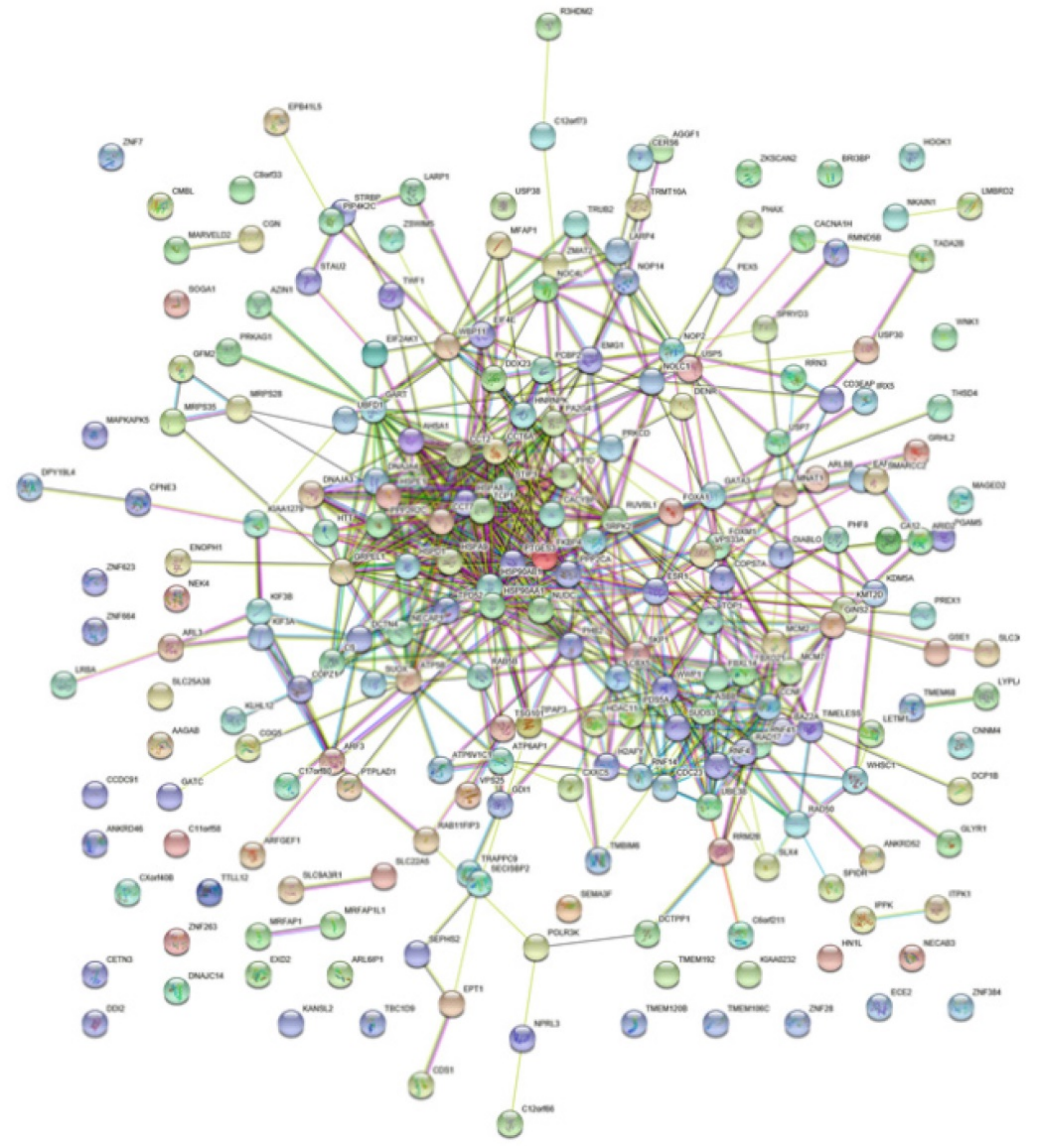
Interactions between different pairs of proteins. Nodes represent various symbols of genes; edges represent protein-protein associations.

A total number of 200 FKBP4 co-expression genes collected from GEPIA were analyzed using the Database for Annotation, Visualization and Integrated Discovery v6.8 (DAVID). The Gene Ontology enrichment analysis comprised three categories: a biological process (BP), a molecular function (MF), and a cellular component (CC). The most valuable 10 pathways of each category were presented in Figure 5A-5C, suggesting that FKBP4 co-expression genes might participate in multiple basic functions including protein folding and binding. The protein-protein interaction (PPI) network was displayed using the String database (Figure 6), and three pairs of co-expressed genes with the highest combined scores (TCP1, CCT2, CCT6A, CCT7, STIP1 and HSP90AB1) were collected from PPI network (Table 1).

### Expression and correlation of co-expressed genes with clinical survival in breast cancer patients

**Figure 7:**
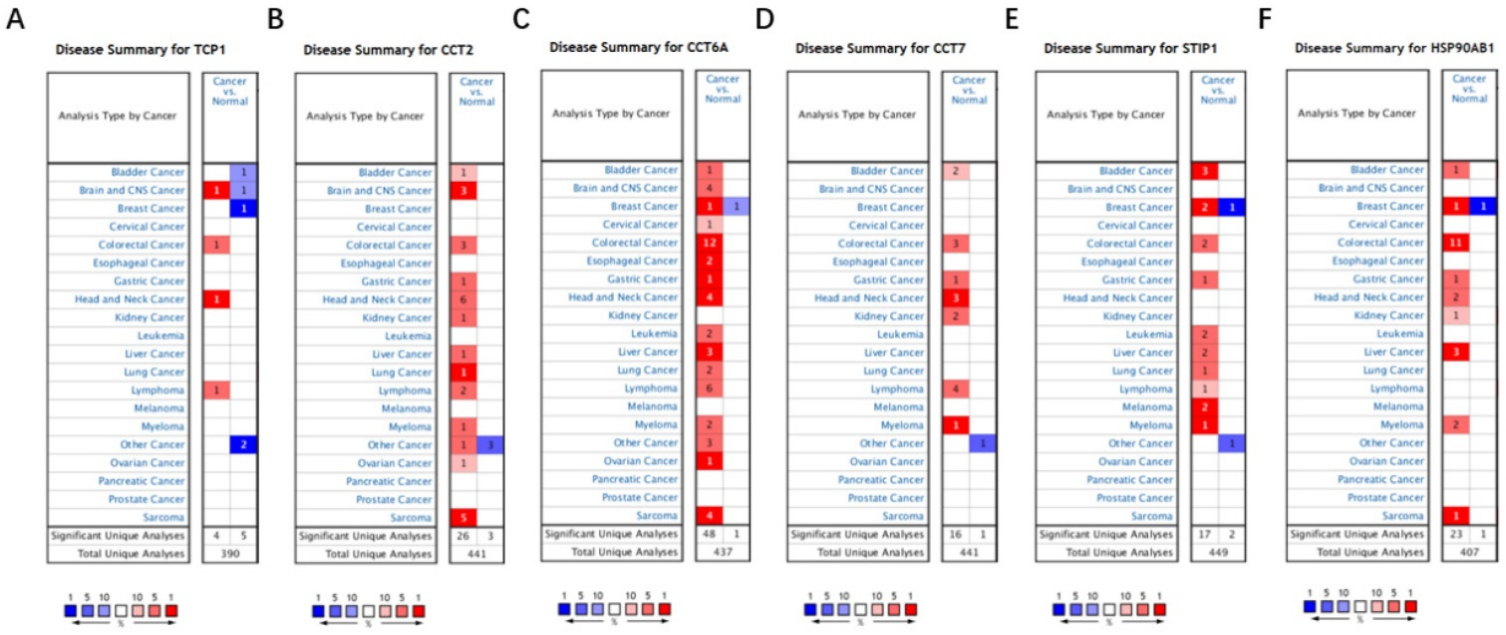
The mRNA expression of FKBP4 co-expressed genes in malignant tumors (Oncomine database). The graph is a representation of the datasets with statistically significant mRNA overexpression (red) or reduced expression (blue) of TCP1, CCT2, CCT6A, CCT7, STIP1 and HSP90AB1 gene (cancer vs normal). Cell color was determined by the best gene rank percentile for the analyses within the cell, and the gene rank was analyzed by percentile of target gene in the top of all genes measured in each research.

**Figure 8:**
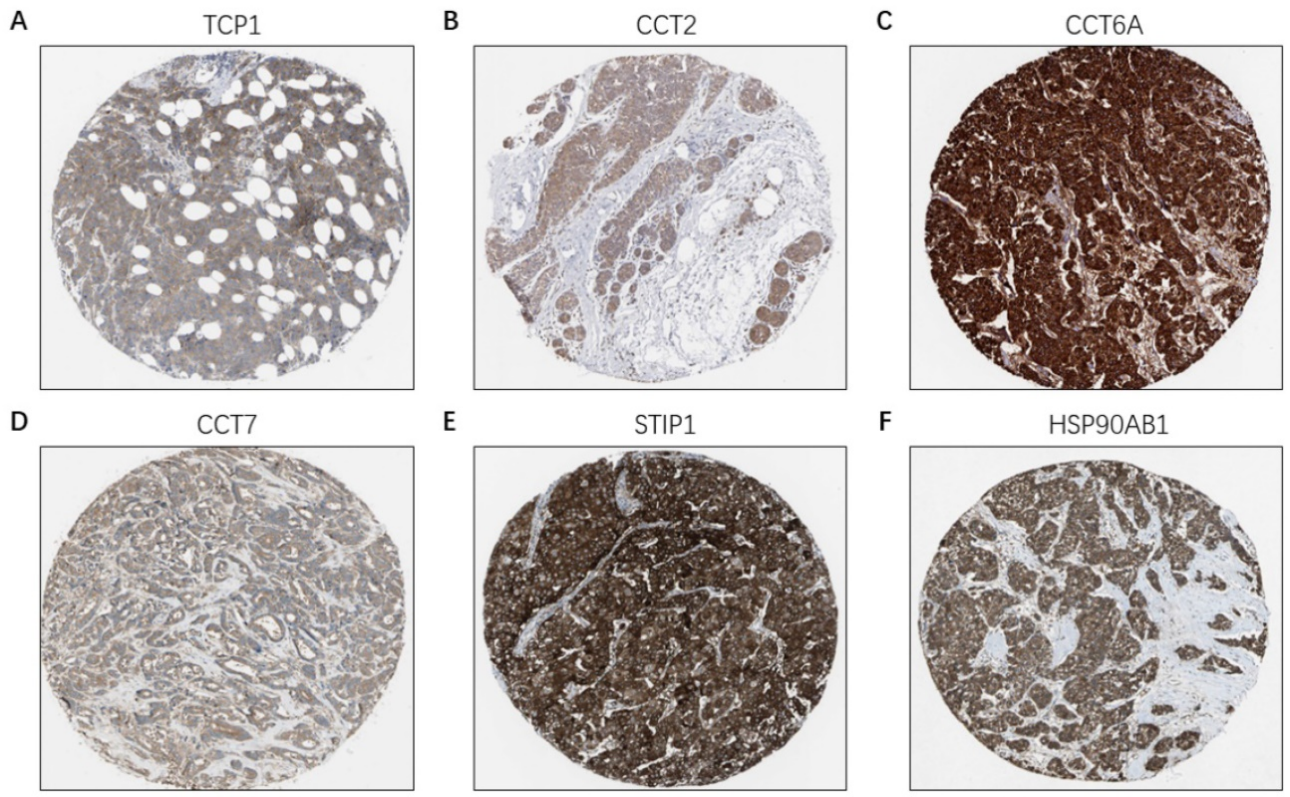
The protein level of FKBP4 co-expressed genes in BC tissues (HPA database). (**A**) TCP1 (Antibody CAB017460) expression in BC tissues. (**B**) CCT2 (Antibody HPA003198) expression in BC tissues. (**C**) CCT6A (Antibody HPA045576) expression in BC tissues. (**D**) CCT7 (Antibody HPA008425) expression in BC tissues. (**E**) STIP1 (Antibody CAB017448) expression in BC tissues. (**F**) HSP90AB1 (Antibody CAB005230) expression in BC tissues.

Based on the Oncomine database, we found that mRNA expressions of TCP1, CCT2, CCT6A, CCT7, STIP1 and HSP90AB1 were significantly upregulated in cancerous samples compared with normal samples in various types of cancer, including BC (Figure 7A-7F). The HPA database indicated that there were high levels of the above-mentioned six co-expressed genes in breast cancer tissues: TCP1 (Antibody CAB017460), CCT2 (Antibody HPA003198), CCT6A (Antibody HPA045576), CCT7 (Antibody HPA008425), STIP1 (Antibody CAB017448), and HSP90AB1 (Antibody CAB005230) (Figure 8A-8F).

**Figure 9:**
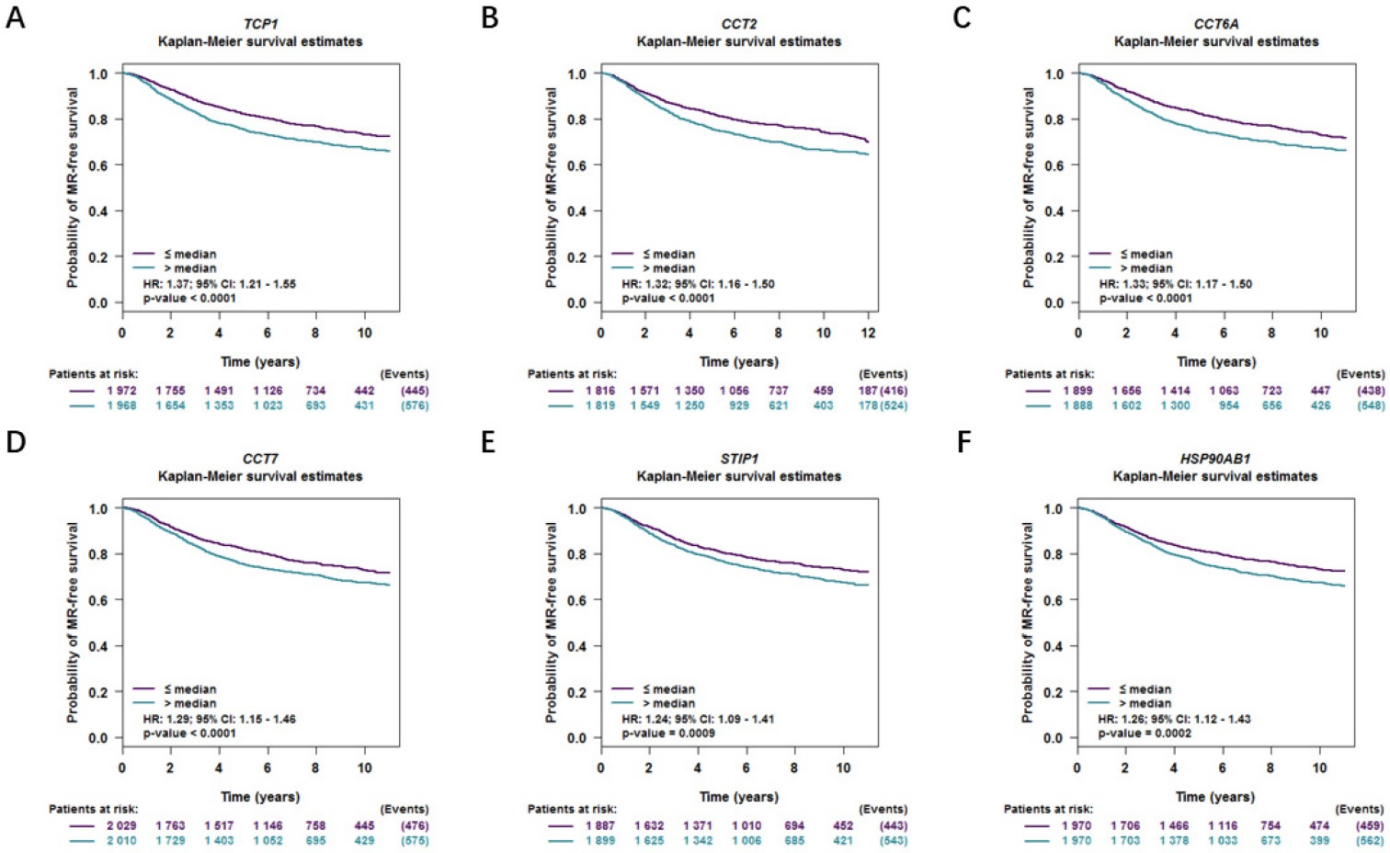
Survival curves in BC patients are plotted for overall subtypes correlated with (**A**) TCP1, (**B**) CCT2, (**C**) CCT6A, (**D**) CCT7, (**E**) STIP1, (**F**) HSP90AB1.

**Figure 10:**
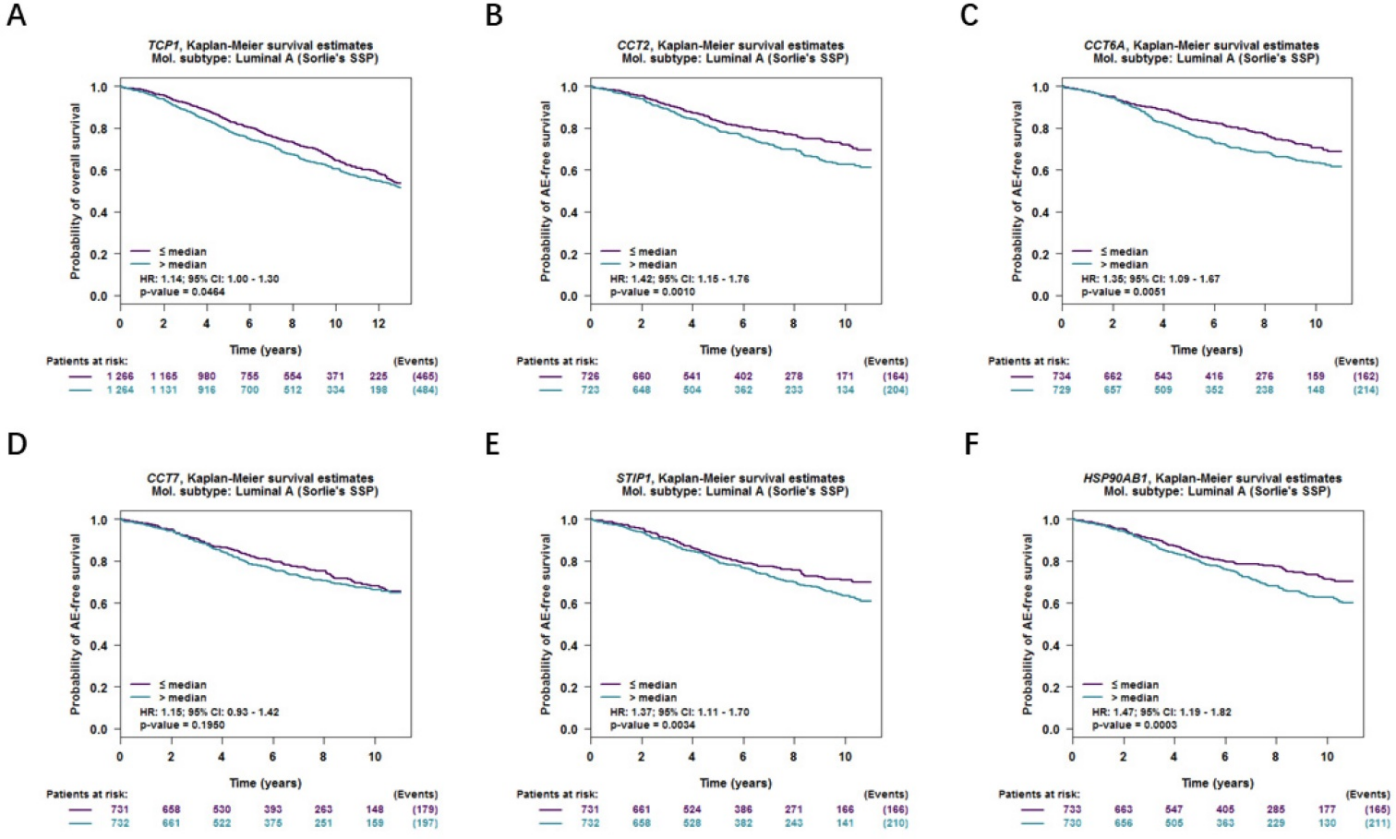
Survival curves in BC patients are plotted for luminal A subtype correlated with (**A**) TCP1, (**B**) CCT2, (**C**) CCT6A, (**D**) CCT7, (**E**) STIP1, (**F**) HSP90AB1.

**Figure 11:**
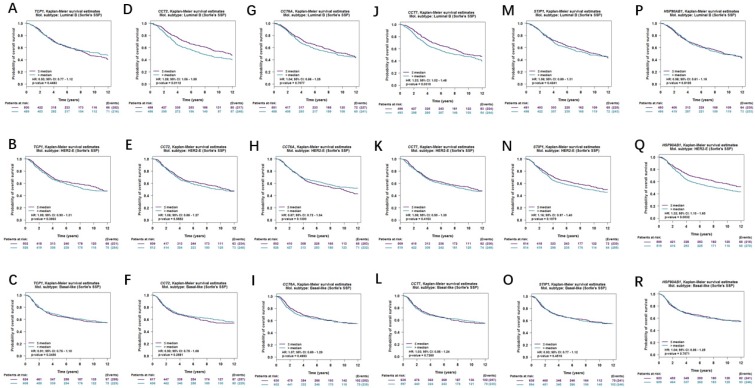
Survival curves in BC patients are plotted for luminal B, HER2 positive and basal-like subtypes correlated with (**A-C**) TCP1, (**D-F**) CCT2, (**G-I**) CCT6A, (**J-L**) CCT7, (**M-O**) STIP1, (**P-R**) HSP90AB1.

Moreover, correlations between co-expressed genes and clinical survival were analyzed by using bc-GenExMiner v4.2, and the Kaplan-Meier curve showed that increased levels of co-expressed genes were all significantly correlated with worse survival in both overall BC (Figure 9A-9F) and LABC (Figure 10A-10F). Meanwhile, six co-expressed genes had no connection with worse survival in luminal B, HER2 positive and basal-like subtypes of BC (Figure 11A-11R). Taken together, upregulated FKBP4 co-expressed genes expression were all correlated with poor survival in LABC patients.

## Discussion

Breast cancer is a leading cause of cancer-related deaths in women aged 40 years and younger^1^. Although in recent years early detection and personalized therapeutics have decreased mortality of BC, discovering novel prognostic indicators are still necessary for improving the prognosis of BC patients. Here, we found that upregulated FKBP4 might play a central role in regulating its co-expressed protein expression in BC.

FK506-binding protein (FKBP) family in *Homo sapiens* (human) genomes has included 18 FKBPs up to date, which can target on various pathways in embryonic development, stress response, cardiac function, cancer tumorigenesis and neuronal function 15. In breast cancer, FKBP5 is the most extensively studied protein among identified human FKBPs, which is demonstrated to interact with HSP90 to affect steroid hormone receptor function 16. In colorectal cancer, silencing FKBP3 has been found to attenuate oxaliplatin resistance by regulation of the PTEN/AKT axis 17.

A growing body of studies observed that FKBP4 expression was also upregulated in different types of cancers, e.g., head and neck cancer, prostate cancer, glioblastoma, ovarian cancer, colon cancer and so forth 3, 6, 18-22. Particularly, data from Yang's study showed that FKBP4 was significantly upregulated in majority of BC cell lines 5, but its expression status and prognostic merit in LABC still remains unclear. In light of these previous studies, we conducted this research to assess the clinical and molecular regulatory importance of FKBP4 in LABC.

In Oncomine and IHC analysis, we illustrated that both mRNA and protein expression of FKBP4 were significantly upregulated in BC tissues than corresponding normal tissues. Then, we detected that FKBP4 high expression in BC significantly correlated with positive nodal status (p=0.0165), ER (p<0.0001) and PR (p=0.0004) status. As for the molecular subtype, the highest expression of FKBP4 was found in luminal B subtype but irrelevant to HER-positive subtype, which suggested FKBP4 might play an indispensable role in ER and PR signaling pathway.

We then used bc-GenExMiner v4.2 database to elucidate that upregulated mRNA expression of FKBP4 was associated with unfavorable survival for all BC patients, and only correlated to worse survival in LABC patients when considering different receptor subtypes. Since ER and PR played pivotal roles in the development and progression of LABC 23, meanwhile FKBP4 chaperonin HSP90 promoted tumor progression by enhancing various oncogenes 24, more researches are warranted to find out whether FKBP4 influences the ER or PR status via HSP90 or they perform collectively toward the prognosis in the BC setting.

Genetic polymorphisms impose vital impact on malignant tumors, but neither Hogewind's research^25^ nor current study revealed that FKBP4 polymorphisms was correlated with breast cancer risk, therefore further researches should be carried out to figure out the prognostic role of FKBP4 polymorphisms in BC patients.

Among the co-expressed genes of FKBP4, a total of six co-expressed genes, TCP1, CCT2, CCT6A, CCT7, STIP1 and HSP90AB1, were finally focused. TCP1, CCT2, CCT6A, CCT7 are all belong to the chaperonin containing TCP1 complex (CCT) [26]and STIP1 is an adaptor protein that coordinates the functions of HSP90AB1^27^. CCT family members overexpression have been reported involved in gene expression and regulation of various carcinomas 28-32. STIP1 and HSP90AB1 are found associated with cell metastasis, apoptosis and other oncogenic functions in human cancer cells 33. In our study, higher expressions of six co-expressed genes were all significantly increased in LABC compared to adjacent healthy controls. Moreover, they were all correlated with a shorter survival time in LABC patients. Therefore, we speculate that these co-expressed genes might also similarly interact with each other via various signaling pathways in LABC. The mechanisms and functions between FKBP4 and co-expressed genes remain elusive and need to be validated, thus promoting the development of efficient therapeutic strategies in LABC in the future.

## Figures and Tables

**Table 1:** Top 20 pairs of co-expressed genes from the PPI network

Node1	Node2	Node1 accession	Node2 accession	Score
TCP1	CCT2	ENSP00000317334	ENSP00000299300	0.999
TCP1	CCT6A	ENSP00000317334	ENSP00000275603	0.999
TCP1	CCT7	ENSP00000317334	ENSP00000258091	0.999
STIP1	HSP90AB1	ENSP00000351646	ENSP00000360609	0.999
STIP1	HSP90AA1	ENSP00000351646	ENSP00000335153	0.999
PTGES3	HSP90AA1	ENSP00000482075	ENSP00000335153	0.999
NOP14	NOC4L	ENSP00000405068	ENSP00000328854	0.999
NOP14	EMG1	ENSP00000405068	ENSP00000470560	0.999
NOC4L	NOP14	ENSP00000328854	ENSP00000405068	0.999
MCM7	GINS2	ENSP00000307288	ENSP00000253462	0.999
MCM7	MCM2	ENSP00000307288	ENSP00000265056	0.999
MCM2	MCM7	ENSP00000265056	ENSP00000307288	0.999
MCM2	GINS2	ENSP00000265056	ENSP00000253462	0.999
HSPE1	HSPD1	ENSP00000233893	ENSP00000373620	0.999
HSPD1	HSPA9	ENSP00000373620	ENSP00000297185	0.999
HSPD1	HSPE1	ENSP00000373620	ENSP00000233893	0.999
HSPA9	GRPEL1	ENSP00000297185	ENSP00000264954	0.999
HSPA9	HSPD1	ENSP00000297185	ENSP00000373620	0.999
HSPA8	HSP90AA1	ENSP00000432083	ENSP00000335153	0.999
HSP90AB1	STIP1	ENSP00000360609	ENSP00000351646	0.999
